# Dopaminergic Modulation of Lateral Amygdala Neuronal Activity: Differential D1 and D2 Receptor Effects on Thalamic and Cortical Afferent Inputs

**DOI:** 10.1093/ijnp/pyv015

**Published:** 2015-03-20

**Authors:** Chun-hui Chang, Anthony A Grace

**Affiliations:** Departments of Neuroscience, Psychiatry, and Psychology, University of Pittsburgh, Pittsburgh, PA 15260 (Drs Chang and Grace).

**Keywords:** amygdala, auditory cortex, auditory thalamus, D1 receptor, D2 receptor, dopamine

## Abstract

**Background:**

In auditory fear conditioning, the lateral nucleus of the amygdala (LA) integrates a conditioned stimulus (CS) from the auditory thalamus (MGN) and the auditory association cortex (Te3) with an aversive unconditioned stimulus. The thalamic input provides a basic version of the CS, while the cortical input provides a processed representation of the stimulus. Dopamine (DA) is released in the LA under heightened arousal during the presentation of the CS.

**Methods:**

In this study we examined how D1 or D2 receptor activation affects LA afferent-driven neuronal firing using *in vivo* extracellular single-unit recordings with local micro-iontophoretic drug application in anesthetized rats. LA neurons that were responsive (~50%) to electrical stimulation in either the MGN or the Te3 were tested by iontophoresis of either the D1 agonist, SKF38393, or the D2 agonist, quinpirole.

**Results:**

We found that most of the LA projection neurons exhibited either facilitatory or attenuating effects (changes in evoked probability >15% relative to baseline) on afferent input by activation of D1 or D2 receptors. In general, it required significantly higher stimulation current to evoke ~50% baseline responses to the cortical input. Activation of the D1 receptor showed no difference in modulation between the thalamic or cortical pathways. On the other hand, activation of the D2 receptor had a stronger inhibitory modulation of the cortical pathway, but a stronger excitatory modulation of the thalamic pathway.

**Conclusions:**

Our results suggest that there is a shift in balance favoring the thalamic pathway in response to DA acting via the D2 receptor.

## Introduction

The neuronal circuitry underlying auditory fear conditioning has been well characterized (Maren, 1999; LeDoux, 2000; Pare et al., 2004). In this behavioral paradigm, an originally neutral tone (conditioned stimulus [CS]) evokes a fear response (conditioned response [CR]) after a few pairing with an aversive mild footshock (unconditioned stimulus [UCS]). The auditory and somatosensory information conveyed by these stimuli converge in the lateral nucleus of the amygdala (LA; Romanski et al., 1993), with the auditory CS entering the LA via the auditory thalamus (medial geniculate nucleus [MGN]) and the auditory association cortex (Te3; Romanski and LeDoux, 1992, 1993). The two auditory pathways convey different aspects of the CS to the LA, with the thalamic input providing basic sensory information regarding the CS, while the cortical input provides a more processed representation of the stimulus (LeDoux, 2000).

Several catecholamines are released under heightened stress or arousal, such as norepinephrine (NE) and dopamine (DA; Pezze and Feldon, 2004; Rodrigues et al., 2009). Indeed, DA is one of the neurotransmitters that potently modulates the underlying states of fear and anxiety (Pezze and Feldon, 2004). Earlier studies have explored extensively the role of DA in fear conditioning. Mild stress and conditioned fear stimuli activate DA neurons in the ventral tegmental area (VTA), as indexed by an increased DA metabolism (Deutch et al., 1985). The presentation of an auditory stimulus previously paired with a footshock increased extracellular DA levels in the amygdala (Suzuki et al., 2002). Systemic administration of either a DA or D1 agonist potentiated conditioned fear (Miczek and Luttinger, 1978; Borowski and Kokkinidis, 1998). Moreover, local D1 and D2 receptors in the amygdala work together to support the formation and expression of conditioned fear (Guarraci et al., 1999, 2000; Nader and LeDoux, 1999a; Greba and Kokkinidis, 2000; Greba et al., 2001). Mechanisms of synaptic plasticity, such as LTP in the amygdala, have been implicated in the storage of the CS-UCS association underlying fear conditioning (Maren, 1999). Physiologically, DA transmission within the amygdala plays an important role in this associative process (Rosenkranz and Grace, 2002).

In an earlier study (Johnson et al., 2011), the authors demonstrated that activation of NE β receptors shifts the balance between the cortical and the thalamic afferent excitation of LA neurons and favors the thalamic pathway, suggesting that under stress and arousal (eg, during fear conditioning) subjects likely attend toward the faster but more primitive input. However, how DA release affects the balance between these two pathways has not been addressed. In this study, we aimed to test the hypothesis that DA in the LA also shifts the neuronal excitability in favor of the thalamic pathway. By using *in vivo* extracellular single unit recordings with local micro-iontophoretic drug application in anesthetized rats, we examined how D1 or D2 receptor activation affected afferent-driven neuronal firing in the LA.

## Methods

### Subjects

A total of 40 Male Sprague-Dawley rats (300–400g; Harlan Laboratories) were used in this study. Rats were housed for at least 5 days in pairs in a temperature (22°C)- and humidity (47%)-controlled facility upon arrival on a 12h light/dark cycle (lights on at 0700h) with food and water available *ad libitum*. Animals were handled in accordance with the guidelines outlined in the United States Public Health Service *Guide for the Care and Use of Laboratory Animals*, and were approved by the Institutional Animal Care and Use Committee of the University of Pittsburgh.

### Electrophysiological Recordings and Iontophoretic Application of Drug

#### Surgery

All recordings were performed on anesthetized rats between 0900 and 1700h as previously described (Chang and Grace, 2013). Rats were anesthetized with 8% chloral hydrate (400mg/kg, i.p.) and placed in a stereotaxic apparatus (David Kopf Instruments); a core body temperature of 37°C was maintained by a temperature-controlled heating pad (FST). Incisions were then made in the scalp to expose the skull. Supplemental doses of chloral hydrate were administered as needed throughout the entire recording session.

#### Electrical Stimulation

A burr hole was drilled into the skull overlying either the MGN (from bregma: anteroposterior [AP], -5.8mm; mediolateral [ML], +3.1mm; dorsoventral [DV], -6.6mm) or the Te3 (from bregma: AP, -5.0mm; ML, +6.5mm; DV, -6.2mm) for the placement of the electrical stimulation electrode. A bipolar concentric electrode (NEX-100X; Rhodes Medical Instruments) was lowered into one of the targets, and stimulation was delivered using a dual-output stimulator (S88; Grass Instruments) at an intensity of 1.0 mA and duration of 0.25 msec at 0.5 Hz in search of evoked responses in the LA (see below).

#### Electrically Evoked Responses

Burr holes were drilled into the skull and the dura was removed in an area overlying the LA (from bregma: AP, -3.0mm; ML, +5.3mm; DV, -6.5 to -9.0mm). Five-barrel microelectrodes (ASI Instruments) were constructed using a vertical microelectrode puller (PE-2; Narishige), and the tip was broken back under microscopic control. The central barrel of the microelectrode was filled with 2% Pontamine sky blue in 2M NaCl with *in situ* impedance of 4–8 MΩ (measured at 1kHz) for electrophysiological recordings. One of the outer barrels was filled with 3M NaCl for automatic current balancing, and the remaining barrels were filled with either the D1 agonist, SKF38393 (20mM in 100mM NaCl, pH = 4.5), or the D2 agonist, quinpirole (10mM in 10mM NaCl, pH = 4.5; Rosenkranz and Grace, 1999; Buffalari and Grace, 2007). The microelectrode was slowly lowered into the LA using a hydraulic microdrive (Model 640; David Kopf Instruments) in search of neurons responsive to MGN or Te3 stimulation. Once a responsive single unit was identified, the stimulation current was adjusted to determine a baseline evoked-spike response probability of ~50% (BL; 20–30 spikes in 50 trials) and the effects of iontophoretic application of either a D1 or D2 agonist on the evoked response were evaluated.

Only single units with response onset latencies <20 msec (presumably monosynaptic) were included for further analyses. These LA neurons showed very minor shifts in latency when the stimulus intensity was increased, yet they showed some range (generally <5 msec) in latency distribution (“jitter”), ruling out antidromic activation. Moreover, all of the neurons reported in this study were putative projection neurons, in that they exhibited very low spontaneous firing rates (<0.5 Hz) and long action potential waveform durations (>2.5 msec; the duration of the action was quantified as the time from the initial change from baseline to the return to baseline) as determined previously (Rosenkranz and Grace, 1999).

#### Iontophoretic Application of Drugs

Because both SKF38393 and quinpirole are weak bases when pH tested, they were held with a (-) retaining current at 10 nA before any ejection currents were tested (E104B; Fintronics). Once a BL-evoked response was obtained, one of the drugs was ejected with a (+) iontophoretic current with successively increasing amplitudes (5, 10, 20, and 40 nA; 50 trials each) to measure the changes in evoked probability of the LA neuron. Putative LA projection neurons were categorized into facilitatory or attenuating D1 or D2 agonists if changes in evoked responses were: (1) unitary in direction; and (2) greater than a 15% change relative to BL at any of the doses applied. Only one drug was applied for each neuron encountered.

### Data Acquisition

Signals from the recording electrode were amplified by a headstage before being fed into a window discriminator/amplifier (1000 gain, 200-16k Hz bandpass; Fintronics Inc.), fed into an audio monitor (AM8; Grass Instruments), and displayed on an oscilloscope (Tektronix) for real-time monitoring. Data were collected using a data acquisition board interface, monitored online, and analyzed offline using computer software (Powerlab, AD instruments).

### Histology

A range of 1–6 neurons was recorded for a single track of search. At the conclusion of each experiment, the microelectrode was replaced to the depth of the neuron recorded, and the location was verified via electrophoretic ejection (BAB-501; Kation Scientific) of Pontamine sky blue dye into the recording site for 30min (−20 μA constant current). If more than one neuron was recorded in a given electrode track, the first and the last neuron encountered were marked at their respective depths, and the recording sites of all neurons were reconstructed according to their relative depth. To verify the placement of the stimulation electrode, a 10 s pulse at 100 μA was administered. Rats were then killed by an overdose of anesthetic (chloral hydrate, additional 400mg/kg, i.p.). All rats were decapitated and their brains were removed, fixed for at least 2 days (8% paraformaldehyde in 0.2M PBS), and cryoprotected (25% sucrose in 0.1M PBS) until saturated. Brains were sectioned (60 μm coronal sections), mounted onto gelatin-chrome alum-coated slides, and stained with a combination of neutral red and cresyl violet for histochemical verification of the stimulating and recording sites.

### Statistics

All data are represented as the mean ± standard error of the mean and differences were tested using analyses of variance (ANOVAs), with the stimulation site (MGN and Te3) and response type (attenuation and facilitation) as the between-subject factors, and current amplitude (BL, 5nA, 10nA, 20nA, 40nA) as the repeated measure. Post hoc comparisons using Fisher’s LSD test were performed for ANOVAs that achieved a significance of *p* < 0.05. All statistics were calculated using SPSS (IBM).

## Results

### Histological Verification

**Figure 1.**
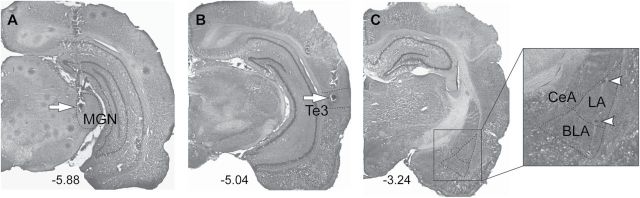
Representative stimulation sites in (A) the MGN and (B) the Te3, as well as a representative recording track in (C) the LA (-5.88, -5.64, and -3.24; anterior-posterior distance to bregma in mm). Arrows indicate the lesion marks at the tips of the stimulating electrodes. Arrowheads point to dye marks of the first and last neurons recorded on the track. BLA, basolateral nucleus of the amygdala; CeA, central nucleus of the amygdala; LA, lateral nucleus of the amygdala; MGN, medial geniculate nucleus;Te3, auditory association cortex.

**Figure 2.**
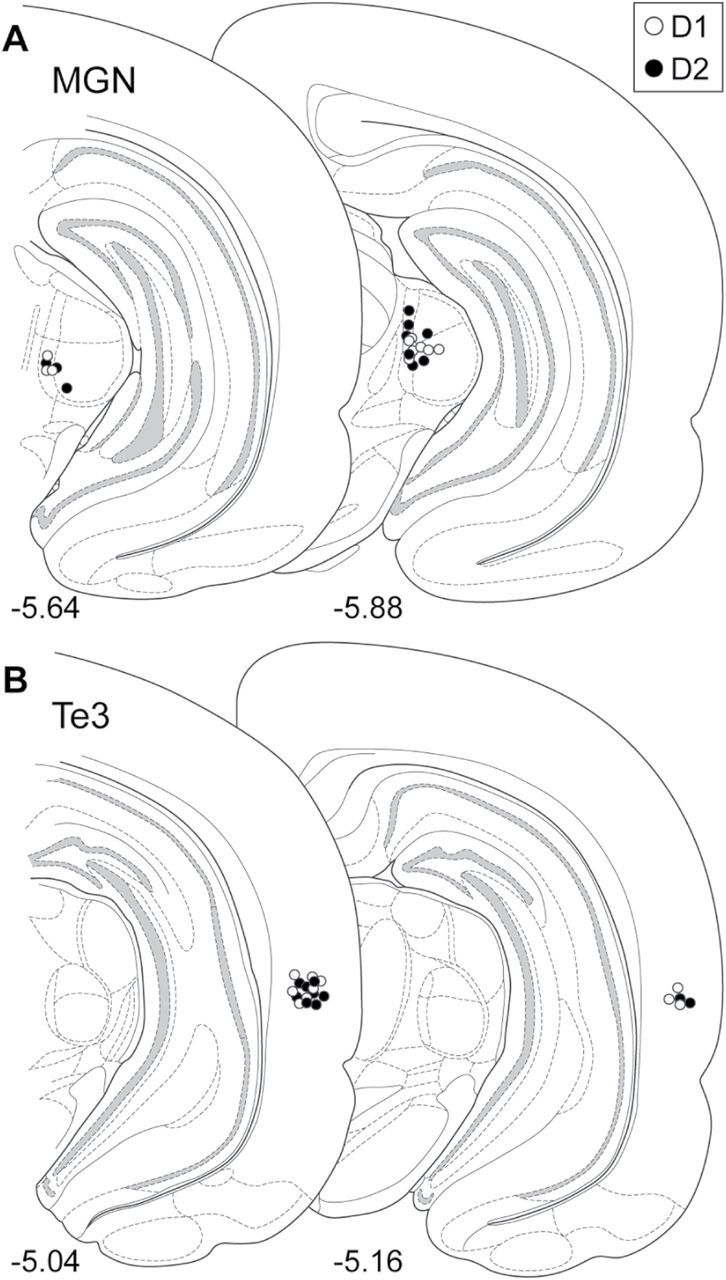
The placements of all the stimulation electrodes in (A) the MGN and (B) the Te3 to examine the D1 agonist (SKF38393; open circle) or D2 agonist (quinpirole; filled circle) modulation of afferent input to putative lateral nucleus of the amygdala projection neurons (-5.64, -5.88, -5.04, and -5.16; anterior-posterior distance to bregma in mm). MGN, medial geniculate nucleus; Te3, auditory association cortex.

Representative stimulation sites in the MGN (Figure 1A) and the Te3 (Figure 1B), as well as a representative recording track in the LA (Figure 1C), are shown in Figure 1. All placements of the stimulation electrodes are summarized in Figure 2. A total of 40 rats were included in this study, with stimulation electrodes in either the MGN (Figure 2A) or the Te3 (Figure 2B) to examine how D1 (open circle) or D2 (filled circle) receptor activation (n = 10 in each condition) modulated evoked responses in the putative LA projection neurons.

### D1 Receptor Activation and Thalamic Versus Cortical Pathway Stimulation

**Figure 3.**
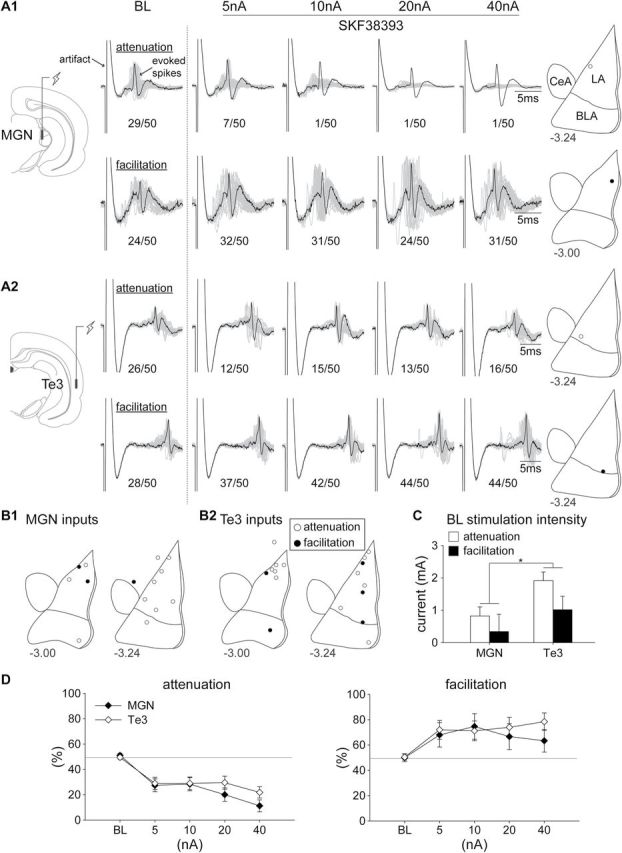
Representative facilitation and attenuation of excitatory inputs in response to D1 receptor activation (SKF38393; step current amplitudes of 5, 10, 20, 40 nA) during stimulation of (A1) the MGN or (A2) the Te3 input. Electrical stimulation currents (mA) were adjusted to evoke a ~50% baseline response (left of the dashed line; 20–30 evoked spikes in 50 trials) for every neuron recorded, with the number indicated underneath each panel (n/50; evoked spikes out of 50 trials in BL and each step current amplitude). Traces of 50 trials (gray and black) were overlaid and aligned to the onset of the electrical stimulation (artifact), with one trace (black) demonstrating the waveform of the neuron. Each neuron is categorized into attenuation or facilitation, based on whether the change in evoked responses is greater than 15% (absolute change in evoked response greater than 7 in 50 trials) relative to BL in any of the doses applied. The location of the representative neuron is labeled on the right panel (-3.00 or -3.24; anterior-posterior distance to bregma in mm; B1 and B2) The distribution of all the neurons recorded (-3.00 or -3.24; anterior-posterior distance to bregma). (C) Higher stimulation currents were required to evoke ~50% BL responses in putative LA projection neurons to the Te3 input compared to the MGN input (*p < 0.05). (D) D1 receptor activation did not differentially affect the facilitation or attenuation of the response to stimulation of the MGN or the Te3 pathways. BL, baseline; BLA, basolateral nucleus of the amygdala; CeA, central nucleus of the amygdala; LA, lateral nucleus of the amygdala; MGN, medial geniculate nucleus; Te3, auditory association cortex.

A total of 33 putative LA projection neurons that responded to MGN or Te3 stimulation were recorded in this experiment. Among the neurons responsive to the MGN input (n = 14), the majority exhibited attenuation of MGN drive in response to D1 receptor activation (79%; n = 11), while the remaining showed facilitation (21%; n = 3). Among the neurons responsive to Te3 input (n = 19), the majority showed attenuation of drive by D1 activation (63%; n = 12), with a minority showing facilitation (26%; n = 5). Two neurons were excluded from further analyses because they showed either bi-directional (Te3, n = 1) or non-responsive (Te3, n = 1) effects to the D1 agonist. Representative facilitatory and attenuating responses to D1 receptor activation (SKF38393; step current amplitudes of 5, 10, 20, 40 nA) are shown in Figure 3A in response to MGN (Figure 3A1) or Te3 (Figure 3A2) stimulation, and the distribution of all the neurons recorded is summarized in Figure 3B. Most of the neurons were located within the LA, with a few in the amygdalostriatal transition area (ASt, n = 3) or the basolateral nucleus of the amygdala (BLA, n = 4).

In general, a greater stimulation current amplitude was required to evoke ~50% BL responses in putative LA projection neurons from the Te3 input compared to the MGN input (Figure 3C). There was a significant main effect of stimulation site [F(1,27) = 5.09, *p* = 0.032]. However, the facilitatory or attenuating effect of D1 receptor activation did not differ when comparing the response evoked from MGN or Te3 pathway stimulation (Figure 3D). The only significant differences were the main effect of response type [F(1,27) = 69.80, *p* < 0.001] and the interaction between response type and current amplitude [F(4,108) = 17.06, *p* < 0.001].

### D2 Receptor Activation, Cortical Input, and the Thalamic Pathway

**Figure 4.**
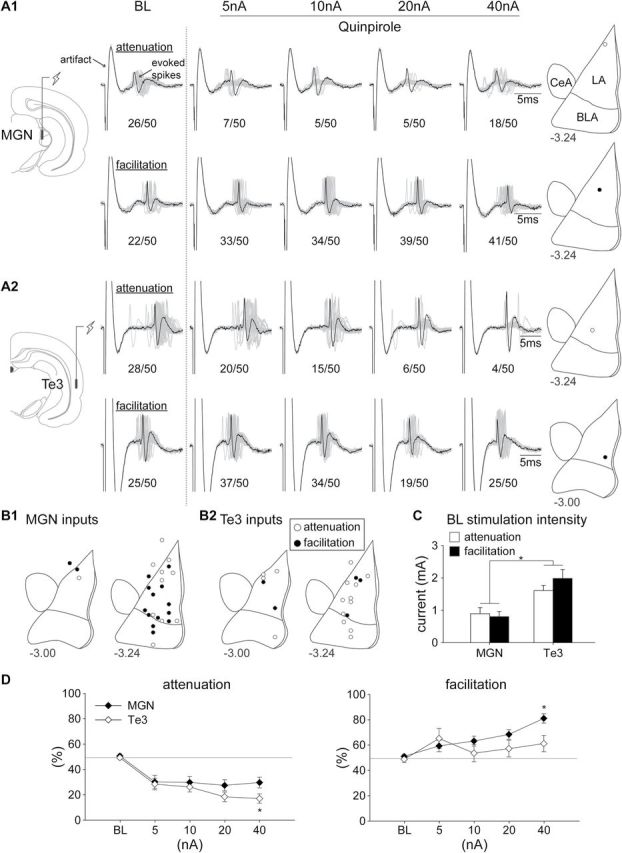
Representative facilitation and attenuation of excitatory inputs in response to D2 receptor activation (quinpirole; step current amplitudes of 5, 10, 20, 40 nA) during stimulation of (A1) the MGN or (A2) the Te3 input. See a detailed description in the legend of Figure 3. (B1 and B2) The distribution of all the neurons recorded. (C) Higher stimulation currents were required to evoke ~50% BL responses in putative LA projection neurons to the Te3 input compared to the MGN input (*p < 0.05). (D) Activation of the D2 receptor exhibited a stronger attenuation of the Te3 pathway (left panel), but a stronger facilitation of the MGN pathway (right panel; *p < 0.05). BL, baseline; BLA, basolateral nucleus of the amygdala; CeA, central nucleus of the amygdala; LA, lateral nucleus of the amygdala; MGN, medial geniculate nucleus; Te3, auditory association cortex.

A total of 54 putative LA projection neurons that responded to MGN or Te3 stimulation were recorded in this experiment. Among the neurons responsive to the MGN input (n = 29), about one third of the neurons showed an attenuation of the evoked response with D2 receptor activation (38%; n = 11), while more than half were facilitated (52%; n = 15). Among the neurons responsive to Te3 input (n = 25), the majority showed an attenuation of the response by the D2 agonist (60%; n = 15), whereas the minority showed facilitation (20%; n = 5). Eight neurons were excluded from further analyses because they showed either bi-directional (MGN, n = 2) or non-responsive (MGN, n = 1; Te3, n = 5) modulatory effects to D2 agonist. Representative facilitatory and attenuating responses to D2 receptor activation (Quinpirole; step current amplitudes of 5, 10, 20, 40 nA) are shown in Figure 4A in response to MGN (Figure 4A1) or Te3 (Figure 4A2) stimulation, and the distribution of all the neurons recorded is summarized in Figure 4B. Most of the neurons were located within the LA, with a few in the ASt (n = 5) or the BLA (n = 7).

In agreement with the experiment above, a higher stimulation current amplitude was required to evoke ~50% BL responses in putative LA projection neurons in response to the Te3 input compared to the MGN input (Figure 4C). There was a significant main effect of stimulation site [F(1,42) = 21.49, *p* < 0.001]. Moreover, activation of the D2 receptor had a stronger inhibitory modulation of the Te3 pathway, but a stronger excitatory modulation of the MGN pathway (Figure 4D). There was a significant main effect of stimulation site [F(1,42) = 4.60; *p* = 0.038], response type [F(1,42) = 89.73, *p* < 0.001], and current amplitude [F(4,168) = 2.45, *p* = 0.048]. ANOVA also revealed a significant interaction between stimulation site and current amplitude [F(4,168) = 3.54, *p* = 0.008] and response type and current amplitude [F(4,168) = 23.08, *p* < 0.001]. A planned comparison between the MGN and the Te3 inputs suggested that at the current amplitude of 40 nA, D2 receptor activation had a significantly stronger excitatory modulation of the MGN input, while the same activation had a significantly stronger inhibitory modulation of the Te3 input (both *p*s < 0.05).

## Discussion

In this study, combined *in vivo* extracellular single-unit recordings and local micro-iontophoretic application of either a D1 or D2 agonist was used to examine the modulatory effect of DA on LA inputs arising from the MGN or the Te3. In general, higher stimulation current amplitudes were required to evoke ~50% BL responses to the Te3 input compared to the MGN input. Supporting our hypothesis, our results suggest that there was a shift in balance favoring the thalamic pathway in response to DA, and this effect was mediated via D2 receptors.

It has been suggested that activation of the thalamic, basic “low road” inputs allows rapid sensory access to the LA in order to initiate rapid, defensive amygdala-dependent fear responses, whereas activation of the cortical, highly-processed “high road” allows refined sensory input to the LA in order to better identify threatening stimuli (LeDoux, 1994, 2000; Johnson et al., 2011). Indeed, fear conditioning using a simple acoustic CS can be mediated by either of these pathways (Romanski and LeDoux, 1992). Despite the fact that activation of either pathway is sufficient to induce fear conditioning, there are differences between the two. For example, single unit recordings in awake and behaving rats suggested that the cortical pathway learns more slowly over trials compared to the thalamic pathway (Quirk et al., 1995, 1997). Moreover, it has been shown that there is correlated change between the amygdala and the thalamus (but not the cortex) during conditioning in a human study, suggesting the importance of the direct thalamic pathway (Morris et al., 1999). In the current study, we found that higher stimulation current amplitudes were required to evoke ~50% BL responses to the Te3 input compared to the MGN input. This result is consistent with and further supports the notion that it requires less effort to engage the direct thalamic pathway, which is critical to generate a fast, and perhaps life-saving, response under threat.

Although we did not use antidromic activation to confirm the cell type, the neurons reported in this study are most likely projection neurons. The majority of the neurons in the BLA are projection neurons (McDonald, 1985), which are more regularly observed using large five-barrel microelectrodes because of their relatively larger soma size, and thus use a higher current density compared to the smaller interneurons (Stone, 1985). Moreover, under the filter setting used (200-16k Hz bandpass), these neurons exhibited long duration action potential waveforms (> 2.5 msec) with very low (< 0.5 Hz) or no spontaneous firing, which is consistent with the characteristics of projection neurons described earlier (Rosenkranz and Grace, 1999). We did encounter some neurons that exhibited characteristics consistent with interneurons, including high spontaneous firing rates (>5 Hz) and short duration waveforms (~1 msec), but these neurons were excluded for data analyses because of the small sample size (total n = 12 from 40 rats). Most of the neurons recorded in this study were located within the LA. We did not exclude the neurons in the ASt or the BLA (n = 19 out of 87) since these neurons exhibited similar firing properties (Clugnet et al., 1990) and the evoked response was consistent with the operational definition of monosynaptic response with latency <20 msec, which is consistent with our previous report (Rosenkranz and Grace, 1999). Nonetheless, our results remain consistent even if the analyses were restricted to neurons within the LA only (data not shown).

Earlier studies examining the impact of DA on LA neuron activity showed inconsistent results. *In vitro* studies suggest that DA enhances the excitability of the LA projection neurons in response to somatic current injections via a postsynaptic effect, in that D1 receptor activation increases excitability and evoked firing, whereas D2 receptor activation increases input resistance (Kroner et al., 2005). On the other hand, *in vivo* studies suggest that DA receptor activation attenuates the firing of the LA projection neurons via a direct inhibition or an indirect action mediated via activation of LA interneurons (Rosenkranz and Grace, 1999). In the current study, we found both excitatory and inhibitory DA modulation on evoked neuron responses, although in general the modulation tended to be attenuating in nature, with D2-mediated facilitation of thalamic inputs representing a smaller proportion of overall responses. Our results also suggest that there was a pathway-specific DA modulation of the responses of putative LA projection neurons that depended on which afferent input was stimulated, and that this difference was dependent on D2 receptors. Thus, D2 receptor activation exhibited a net stronger excitatory modulation of the MGN pathway and a stronger inhibitory modulation of the Te3 pathway. In contrast to *in vitro* studies, which are more effective at examining transmitter effects on isolated systems, *in vivo* extracellular recordings best preserve the entire neural circuitry and thus reflect the overall impact of local DA modulation. Moreover, local micro-iontophoretic application of either the D1 or D2 agonist would better represent the impact of fast DA action upon the local afferent terminals, ruling out the potential confound that would result from systemic drug administration acting on afferent neuron somata or via D2 autoreceptors in the VTA as suggested earlier (Nader and LeDoux, 1999b; Pezze and Feldon, 2004). Thus, our results provide strong evidence regarding how DA release can shift the balance of information flow toward the direct and fast thalamic pathway under stress and arousal. In our experience, we have not observed evidence for short- or long-term plasticity induced at the frequency (0.5 Hz) of stimulation used in the current study (see Gill and Grace, 2011). Of course, we cannot rule out the possibility, however unlikely, that there may have been some plasticity induced beyond the transient modulation observed in the presence of the D2 agonist. It is worth noting that although there is a shift toward the subcortical pathway under the influence of NE (Johnson et al., 2011) and DA (this study) that mimics the situation that would be present when the animal is under threat, the two pathways are both necessary and critical for survival, especially when the animal needs to evaluate a more complex stimulus pattern (Jarrell et al., 1987; LeDoux, 2000; Johnson et al., 2011), which is more likely to occur in the natural environment.

For VTA DA neurons to properly respond to a salient stimulus, the DA neurons must be spontaneously firing (Floresco et al., 2003; Lodge and Grace, 2006; Sesack and Grace, 2010). The proportion of DA neurons firing spontaneously depends heavily on the state of the animal. Acute stress induces a pronounced activation of the VTA DA system (Valenti et al., 2011), which further supports a condition wherein the organism is more likely to switch to the fast and direct thalamic pathway under threat. Although acute stress is in general considered beneficial in generating fast, coping, “flight-or-fight” behaviors, chronic stress is very likely to induce maladaptive effects on the brain (McEwen, 2007). Chronic or repeated stress triggers synaptic remodeling in the amygdala (Roozendaal et al., 2009) and an attenuation in VTA DA neuron tonic activity (Chang and Grace, 2014). Under such conditions, the slower, more evaluative cortical pathway would predominate. However, if this situation progresses to the point where the organism becomes ruminative instead of proactive, as is proposed to occur in depression (Belzung et al., 2014), this would be highly deleterious to survival. Thus, further work on how acute and chronic stress regulates the balance between the cortical and the thalamic inputs on LA neuronal excitability and how this is dependent on DA and/or NE modulation is a critical next step to advance our understanding of adaptive and maladaptive fear regulation.

## Statement of Interest

Drs Chang and Grace report no biomedical financial interests or direct conflicts of interest. Dr Grace receives funds from several organizations, including grant support from Lundbeck and Lilly, and has honoraria/consultant arrangements with Johnson & Johnson, Pfizer, GSK, Merck, Takeda, Otsuka, Lundbeck, Lilly, Roche, Asubio, and Abbott. None of the data reported in this manuscript are related to the funding or consulting performed for these companies.
